# Acute endothelial response to testosterone gel administration in men with severe hypogonadism and its relationship to androgen receptor polymorphism: a pilot study

**DOI:** 10.1007/s40618-015-0325-4

**Published:** 2015-07-11

**Authors:** D. Francomano, G. Fattorini, D. Gianfrilli, D. Paoli, P. Sgrò, A. Radicioni, F. Romanelli, L. Di Luigi, L. Gandini, A. Lenzi, A. Aversa

**Affiliations:** Department of Experimental Medicine, Endocrinology and Food and Science Section, “Sapienza” University of Rome, 00161 Rome, Italy; Department of Movement, Human and Health Sciences, University “Foro Italico” of Rome, 00135 Rome, Italy

**Keywords:** Male hypogonadism, Cardiovascular, Reactive hyperemia index, CAG repeat, Treatment

## Abstract

**Purpose:**

Testosterone (T) exerts different effects on the cardiovascular system. Despite this knowledge, the acute vascular effect of androgen remains still poorly understood.

**Methods:**

We investigated the acute effects of T on vascular function in ten men (18–40 years age) with hypogonadism and severe hypotestosteronemia [serum total testosterone (TT) = 0.6 ± 0.3 ng/mL]. In a 4-day double-blind, randomized, placebo-controlled crossover study, we administered 80 mg daily dose of transdermal-T gel (TG) and evaluated endothelial variations with Endopat2000 (reactive hyperemia index, RHI and the augmentation index, AI); also, CAG repeat polymorphism in exon 1 of the androgen receptor gene was investigated.

**Results:**

After TG administration, RHI significantly improved at 4 h (*p* < 0.05), while AI improvement was recorded at 4 and 96 h, also when adjusted for heart rate (AI@75; *p* < 0.01 and *p* < 0.001, respectively). Direct relationships between ΔT, ΔDHT and ΔRHI variations (*r* = 0.37, *p* < 0.01; *r* = 0.17, *p* < 0.05, respectively) as well as between “CAG repeats” length and ΔLnRHI at 96 h (*p* < 0.03, *r*^2^ = 0.47) were found. An inverse relationship between ΔT and ΔAI (*p* < 0.01, *r* = −0.35) and ΔAI@75 (*p* < 0.01, *r* = −0.38) were found.

**Conclusion:**

Administration of TG causes an acute vasodilation and improves arterial stiffness probably due to non-genomic actions of T. Endothelial vasodilatory response was more pronounced depending on higher plasma TT and DHT levels attained. Clinical implications in elderly frail populations are discussed.

## Introduction

The endothelium is a single layer of cells that line the luminal surface of blood vessels. In a normal physiologic state, healthy endothelium serves as an anticoagulant membrane, exerting predominantly fibrinolytic, anticoagulant and anti-aggregation effects [1]. The vascular endothelium also plays an obligatory role in vasodilation by the action of endothelial-derived nitric oxide (NO). The presence of different cardiovascular risk factors (CRFs), such as aging, smoking, hypertension, dyslipidemia, diabetes, obesity and some less traditional factors (e.g., inflammation, hypoxia, oxidative stress and hyperhomocysteinemia), is known to cause endothelial dysfunction (EDys) [2]. EDys frequently occurs in acute coronary syndromes [3], heart failure and erectile dysfunction [4]. EDys is characterized by significant modifications in the physiological and biochemical parameters in the endothelium. These include: vascular stiffness, increased vascular tone, production of inflammatory cytokines, increased permeability, susceptibility to invasion of immunocytes, decrease in endothelial cell growth and dysregulation of fibrinolytic factors.

Exploration of the brachial artery flow-mediated dilation (FMD) capability is the most commonly used technique for investigation of endothelial function. Flow-mediated dilation serves as an index of NO-mediated endothelium-dependent vasodilator function [5, 6]. Recently, an alternative method, reactive hyperemia peripheral arterial tonometry (PAT), has been used to indirectly identify patients with coronary endothelial dysfunction by measuring pulse volume changes at the fingertips after an occlusion of the brachial artery of the dominant arm. This method showed a significant correlation with FMD (*r* = 0.55, *p* < 0.0001) [7]. The PAT evaluates the amplitude of the arterial pulse wave at the fingertip; this is a non-invasive, operator-independent technique which allows plethysmographic recording of the pulse wave amplitude that corresponds to the modifications of the pulsatile blood volume. Reactive hyperemia has been showed to be an adequate surrogate marker useful for monitoring the changes of vascular function; in the Framingham cohort, PAT measurement proved to be closely related with the cardiovascular risk factors. In addition, a number of other studies showed a relation between coronary EDys, assessed through invasive methods and results obtained through PAT [8]. EDys is a systemic process and the assessment of the functional endothelium-dependent capability of the peripheral arteries represents a chance to examine in depth the functional status of the whole circulatory system [9].

There are pre-clinical and clinical scientific evidences that correlate EDys with androgen deficiency [10]. Several in vitro studies examined the physiological mechanisms of the androgen-mediated vasodilation in various animal and human blood vessels, showing that the relaxing effects of T and 5α-DHT on vascular and non-vascular smooth muscles might probably be due to inhibition of the L-type calcium channels [11]. In some studies, 5α-DHT proved to be more powerful than T in eliciting relaxation of KCl-induced contractions. T, at nanomolar concentrations, showed to be a potent L-type voltage-operated calcium channels (L-VOCCs) antagonist ascribable to a 5α-DHT selective block. Akishita et al. in a cohort of 187 men demonstrated that the percentage of FMD in the highest quartile of free testosterone was 1.7-fold higher than that in the lowest quartile [12]. In particular, serum total and free testosterone (TT and FT, respectively) and dehydroepiandrosterone sulfate (DHEA-S) concentrations were significantly correlated with the percentage of FMD independently from other risk factors such as age, body mass index (BMI), hypertension, dyslipidemia and diabetes. Foresta et al. showed that transdermal-T administration was able to increase the number of circulating endothelial progenitor cells [13]. Jones et al. reported that T treatment in male patients with cardiovascular disease was related with an improvement of vascular reactivity, suggesting that this can be true only for pathologic blood vessels [14]. We recently demonstrated that long-term T administration in obese hypogonadal men is able to improve metabolic parameters as well as endothelial function and that a 6-month withdrawal reverts most of the beneficial effects [15].

Despite these studies, the effects of T on vascular reactivity may be also dependent on the variable number of CAG repeats in exon 1, which encodes a polyglutamine tract in the amino-terminal domain of the androgen receptor (AR) present in human vessels of healthy subjects [16]. The present study was designed to investigate the acute effects of T administration on vascular parameters in young severe hypogonadal men of any origin, and the possible relationship of the vascular response with the polymorphism of AR.

## Materials and methods

### Patients

Ten men affected by severe hypotestosteronemia, after a pharmacologic T treatment washout period of 4 weeks, were enrolled. The main features of the enrolled subjects are reported in Table 1. All the subjects were non-smoking and normal weight. Patients with primary hypogonadism were affected by Klinefelter’s syndrome (*n* = 1) and bilateral orchiectomy because of testicular cancer (*n* = 3), while six out of ten were affected by secondary hypogonadism.

**Table 1:** Baseline demographic features of enrolled subjects

	*n* = 10
Age (years)	31 ± 9
Systolic blood pressure (mmHg)	103 ± 10
Diastolic blood pressure (mmHg)	70 ± 13
Body mass index (BMI, kg/m^2^)	22 ± 3
Subjects with primary hypogonadism	4
Subjects with secondary hypogonadism	6

Inclusion criteria were age greater than 18 years and serum total testosterone (TT) level <1 ng/mL (3.5 nmol/L) at time 0, confirmed at two different times. Exclusion criteria were the following: past events of cardiomyopathy, stroke and deep venous thrombosis; change of therapy in the last 6 months; prostate or breast cancer; high values of PSA (adjusted for age); digital rectal examination suggestive of prostate cancer; severe obstructive symptoms of benign prostate hyperplasia (IPSS > 18); hematocrit level >52 % at time 0; previous or present signs of chronic liver or kidney disease, hematological disorders, hyperprolactinemia or other endocrine disorders (i.e., “empty sella syndrome”, MR-detected pituitary masses); other conditions hazardous to the health of patients. Written informed consent was obtained before commencement of the study, according to Protocol and Good Clinical Practice (GCP) on the conducting and monitoring of clinical studies and approved by our internal review board.

### Study design and main outcome measures

This randomized, double-blind, crossover, placebo-controlled study used a fixed dose (4 g at 2 %) of transdermal-T gel (TG; 80 mg testosterone daily—Tostrex^®^) or as an alternative an equivalent dose of placebo gel for four consecutive days. Each subject received the same information about how to correctly use the gel preparation that should have been applied in the early morning (between 8:00 and 9:00 a.m.), after shower or bathing, on hairless skin.

Each subject underwent endothelial function assessment through peripheral arterial tonometry (PAT; EndoPAT2000, Itamar Medical, Caesarea, Israel) according to previously published procedure [17] and blood sample at times 0, +4 and +96 h (the latter before gel administration) were investigated. In each blood sample, the following substances were dosed: TT, DHT, FT, 17β-estradiol (E2) and sex hormone binding globulin (SHBG). At the end of each treatment, all patients were shifted to an alternative treatment after a washout period of 7 days to avoid any accumulation effect. Blood samples were centrifuged and immediately frozen at −20 °C. Each test was performed in duplicate. TT and E2 were measured through chemiluminescence microparticle immunoassay (CMIA, Architect System, Abbott Laboratories, IL, USA), with a detection limit of 0.28 nmol/L and 10 pg/mL, respectively (intra- and interassay coefficients of variation for TT: 2.1 and 3.6 % at 10.08 nmol/L), with reference values 2.8–11 ng/mL (T) and 25–107 pg/mL (E2). Serum concentrations of SHBG were measured through radioimmunoassay (Radim S.p.A., Pomezia, Roma, Italia), with detection limit of 0.26 nmol/L, intra- and interassay coefficients of variation 2.8 and 4.1 % at 26.4 nmol/L and reference values 9–54 nmol/L. FT concentrations were assessed through radioimmunometric assay (RIA) (DIA Source ImmunoAssays S.A.—Louvain-la-Neuve, Belgio), with detection limit of 0.13 pg/mL, intra- and interassay coefficients of variation 5.7 and 6.7 % at 10.89 pg/mL and reference values 8.9–42.5 pg/mL. Serum concentrations of DHT were measured by radioimmunoassay (RIA) using commercial RIA kits (Diagnostic System Laboratory, Webster, TX, USA), with an intra- and interassay coefficient of variation of 3.7 and 6.9 %.

The polyglutamine and polyglycine segment polymorphism of the AR gene (CAG and GGC repeats) was also performed in all subjects. Genomic DNA extraction and analysis of the length of the polymorphic fragments were performed according to a previously published procedure [18].

Primary end points were variations from baseline of the hyperemic response (RHI) as calculated by fingertip peripheral arterial tonometry (EndoPAT2000) and of the augmentation index (AI).

Also, the study of androgen receptor polymorphisms, via measurement of “CAG repeats” length, was performed to evaluate the possible relationship with vascular responses.

### Statistical analysis

All results are reported as mean ± standard error (SE). Statistical differences for single between-group comparisons were studied using *t* tests for non-paired data. Multiple regression analysis was performed to assess values of RHI and AI in comparison with values of TT and FT (at times 0, +4 and +96 h) and values of ΔRHI and ΔAI in comparison with values of ΔTT and ΔFT; the same statistical analysis was performed also for values of length of sequence of CAG triplets (CAG repeats) in comparison with values of RHI and the natural logarithm (LnRHI), at times 0, +4 h and +96 h. Statistical analysis was performed using software SPSS 17.0 (SPSS Inc., Chicago, IL, USA). A *p* value <0.05 was considered to be statistically significant.

## Results

**Fig. 1:**
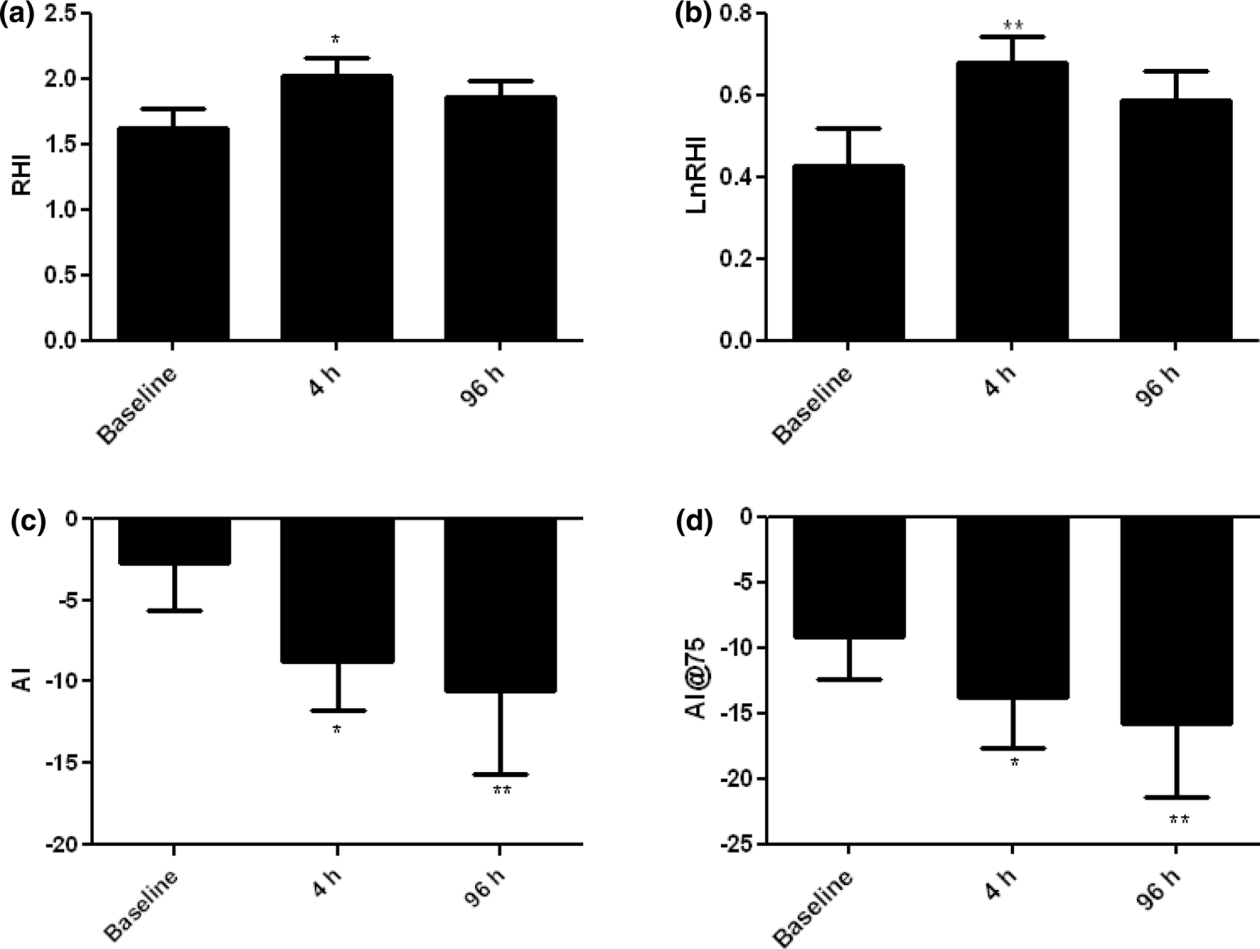
Reactive hyperemia index (RHI) and augmentation index (AI) in the T treatment arm (*p < 0.01; **p < 0.001)

**Fig. 2:**
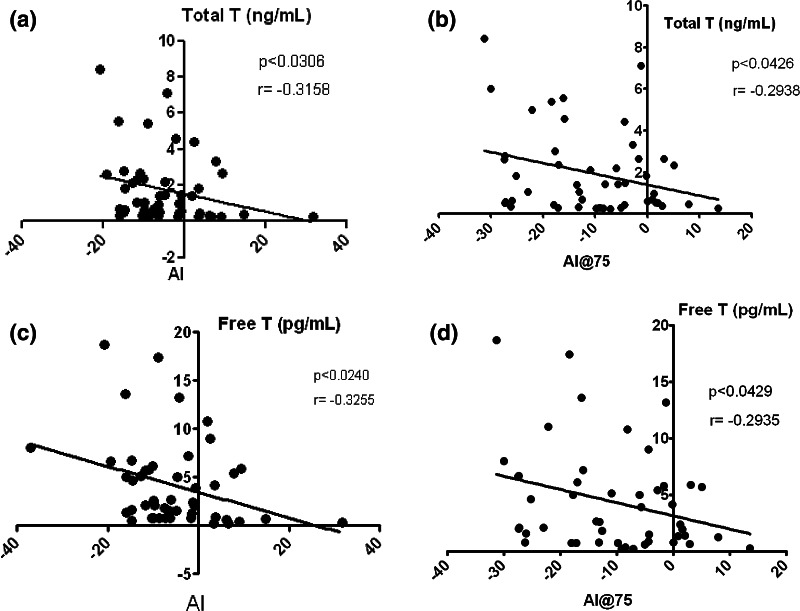
Correlations between augmentation index (AI) and androgen levels

4 and 96 h after starting treatment with TG, 80 mg daily, serum TT, FT and DHT levels showed a statistically significant increase compared to the basal values for all subjects, (*p* < 0.0001, *p* < 0.001 and *p* < 0.001, respectively; Table 2). No statistically significant change in E2 and SHBG levels was found (Table 2). RHI and LnRHI showed a statistically significant increase (mean ± SE) only at time +4 h compared to the basal value (*p* < 0.01 and *p* < 0.001 Fig. 1a, b, respectively). Also, AI and AI@75 values showed a statistically significant reduction compared to baseline at +4 h (*p* < 0.01) and +96 h (*p* < 0.01 and *p* < 0.001, Fig. 1c, d respectively). An inverse correlation was found between TT and AI (*p* < 0.03; *r* = −0.31; Fig. 2a) and AI@75 (*p* < 0.04; *r* = −0.29; Fig. 2b); similarly, an inverse correlation was found between FT and AI (*p* < 0.02; *r* = −0.32; Fig. 2c) and AI@75 (*p* < 0.04; *r* = −0.29; Fig. 2d). No hormonal and vascular differences were found after placebo treatment.

**Table 2:** Differences in hormonal levels in different treatment groups

	Testosterone treatment arm			*p*	Placebo arm			*p*
	Time 0	Time +4 h	Time +96 h		Time 0	Time +4 h	Time +96 h	
Total T (ng/mL)	0.71 ± 0.48	4.55 ± 2.13***	1.96 ± 0.68**	*p* < 0.0001*** *p* < 0.001**	0.58 ± 0.43	0.55 ± 0.26	0.55 ± 0.46	ns
Free T (pg/mL)	1.6 ± 1.8	10.1 ± 5.2***	5.2 ± 2.6**	*p* < 0.0001*** *p* < 0.001**	1.54 ± 1.3	1.2 ± 0.75	0.72 ± 0.51	ns
DHT (pg/mL)	305 ± 140	553 ± 105**	410 ± 141*	*p* < 0.001** *p* < 0.01*	330 ± 150	312 ± 138	328 ± 152	ns
E2 (pg/mL)	11.1 ± 1.7	12.1 ± 4.8	14.6 ± 4.5	ns	10.8 ± 2.05	10.8 ± 1.6	10.3 ± 10.5	ns
SHBG (nMol/L)	34.6 ± 12.7	35.7 ± 12.8	33.7 ± 12.7	ns	35.5 ± 7.1	32.7 ± 12.8	32.6 ± 7.5	ns

**Fig. 3:**
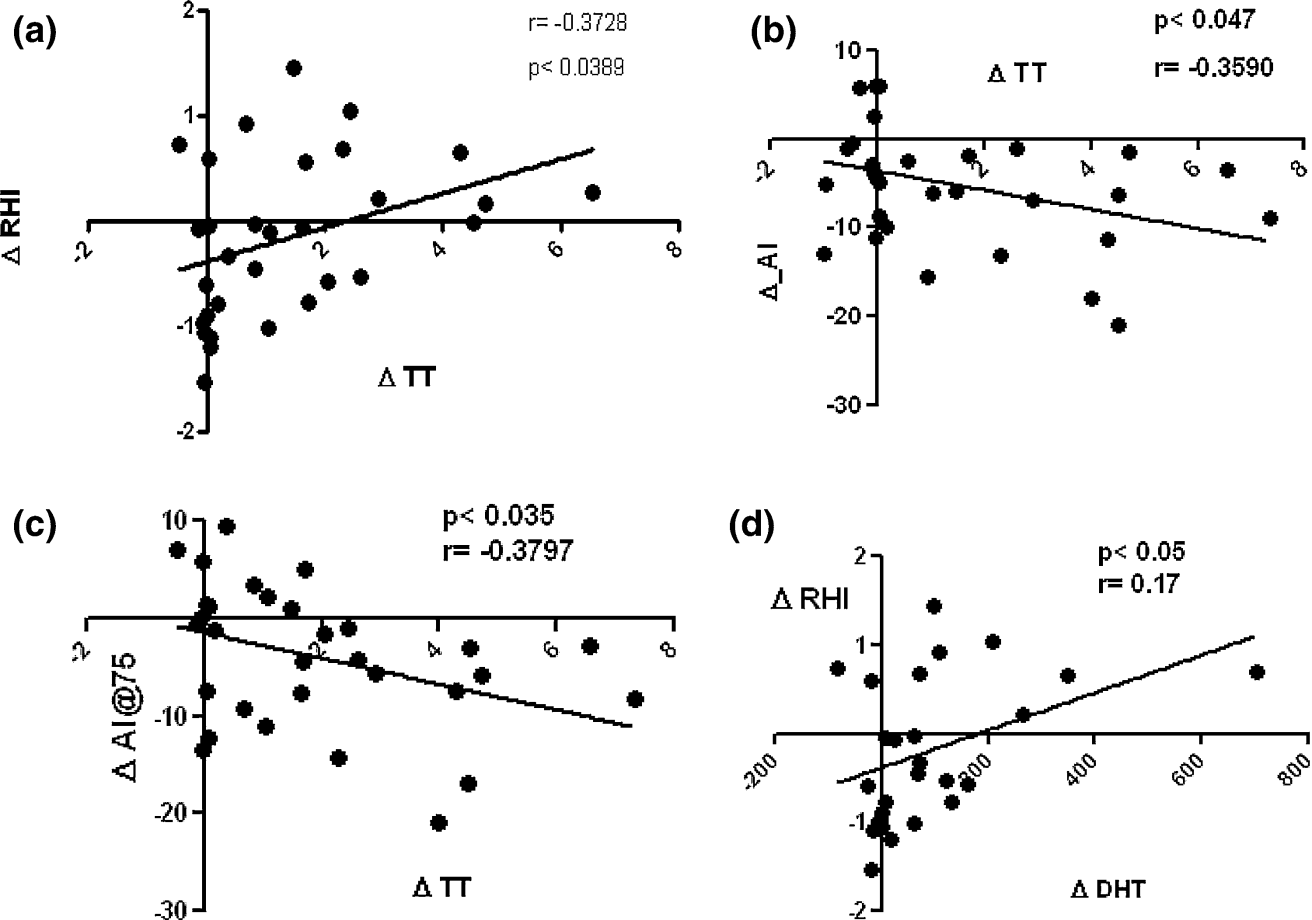
Correlations between TT and DHT vs. variations of EndoPAT parameters

**Fig. 4:**
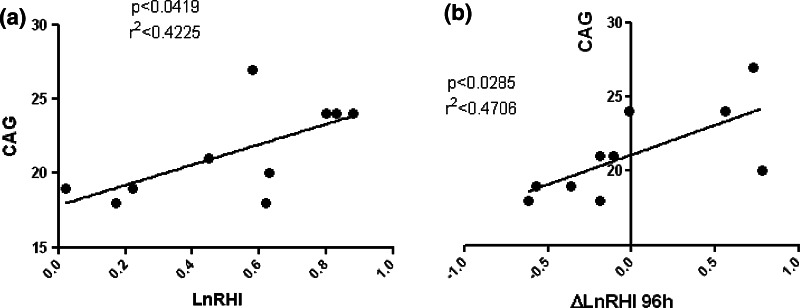
Correlations between “CAG repeats” and variations of natural logarithm of RHI

Interestingly, a direct correlation was found between ΔTT and ΔRHI (*p* < 0.04; *r* = 0.37; Fig. 3a). Accordingly, an inverse correlation was found between ΔTT and ΔAI (*p* < 0.05; *r* = −0.35; Fig. 3b), ever after normalization for heart rate, ΔAI@75 (*p* < 0.03; *r* = −0.38; Fig. 3c), and between ΔDHT and ΔRHI (*p* < 0.005; *r* = −0.17; Fig. 3d). Furthermore, a direct correlation was found between “CAG repeats” length and LnRHI (Fig. 4a) and only after +96 h between “CAG repeats” and ΔLnRHI (*p* < 0.03; *r*^2^ = 0.47) (Fig. 4b). No serious adverse events were reported.

## Discussion

This is the first study to our best knowledge that evaluated the acute (+4 and 96 h) vascular effects of T administration in young hypogonadal men through non-invasive digital peripheral arterial tonometry (PAT). We found that the administration of a fixed dose of 80 mg TG promptly restored T plasma levels and improved endothelial function, depending also on AR CAG repeat lengths. Our results show a statistically significant acute improvement of endothelial function as evaluated by PAT, with a concomitant reduction of the arterial stiffness. Indeed, Webb et al. had previously demonstrated similar acute effects after intracoronary administration of physiological T concentrations, by inducing coronary artery dilatation and increases in coronary blood flow in men with established coronary artery disease (CAD). Despite that in vitro and in vivo studies in animals support the hypothesis that T can lead to endothelium-independent vasodilation mechanisms through a direct interaction with K^+^ or Ca^2+^ channels, the results of that study were pivotal to hypothesize an indirect, endothelium-dependent mechanism of action of T [19].

It has been suggested that E2 has vasodilator properties through NO endothelial production and direct smooth muscle relaxation [20], but also T has been shown to cause vasodilatation by direct interaction with ion channels within the vascular smooth muscle cell membrane [21], i.e., through inhibition of l-type voltage-gated calcium channels (VGCCs) [11]. This evidence has been noticed in clinical trials reporting reduction in blood pressure occurring in hypogonadal man treated with T [22]. The absence of E2 variations observed in our study seems to exclude the involvement of estrogens in the acute vasodilatory effects reported by T administration. Thus, these latter effects might be directly due to T-induced non-genomic effects on arterial vasodilation that are in accordance with the results of several preclinical and clinical studies. Additionally, the direct relationship with CAG repeats found in our results after 96 h could explain some genomic (late) effects of T replacement therapy, in accordance with the potential chronic therapeutic protective effect on CAD as shown in previous studies [19].

Two distinct and large bodies of literature exist on resilience that are of potential interest for surgical outcomes: first, literature on the impact of resilience on surgical recovery and wound healing; second, literature on biomarkers for resilience, which largely focuses on neuropeptide Y, dehydroepiandrosterone and T [23]. By identifying surgical resilience, there is potential for utilizing these biomarkers as prognostic indicators of likely recovery trajectories from surgery, which in turn complement individualized peri-operative management. As such, T represents one of the most prominent biomarkers and its short-term administration may represent a challenge for some categories of patients after surgery. Yaron et al. firstly demonstrated that low T serum levels were directly correlated with increased arterial stiffness and that restoring normal T serum levels with 1 % transdermal-T (25–50 mg daily) was able to improve endothelial function after 48 h and 90 days [24]. Hu et al. reported that middle-aged male patients with CAD presented lower levels of serum T and that testosterone level was negatively correlated with the severity of coronary artery stenosis; patients with acute myocardial infarction were found to have the lowest T levels (365.3 ng/dL) in the series. T levels were also found to be independent predictors for CAD (odds ratio 0.311, 95 % confidence interval 0.174–0.512) [25]. Atish et al. also found that long-term testosterone replacement therapy could decrease angina threshold and promote plaque stability in men. These results suggested that lower T level could be involved in the pathogenesis of CAD and exogenous T replacement therapy may be a potential therapeutic approach for CAD [26]. The results from our study suggest that T supplementation could be a potential approach for both patients after surgery resilience or myocardial infarction; a short challenge of T could improve their potential vasodilatory responses to conventional treatments and their recovery.

With regard to T abuse, we might hypothesize that a rapid effect of exogenous T administration on the vascular system could also contribute to an acute muscular performance improvement in athletes abusing a single TG administration during prolonged aerobic competition.

The present study has some limitations. The number of patients studied is low, so that major conclusions on CAG number repeats and its relationship with vascular parameters cannot be obtained. Nevertheless, severe young hypogonadal patients are difficult to enroll because of the low incidence of disease, but they represent a population that is often free from potential confounding factors, i.e., concomitant drug assumption. We are aware that increasing the number of subjects studied could have a different impact on the present findings.

## Conclusions

We demonstrated for the first time that acute TG administration in severe hypogonadal men improves arterial vasodilatory responses in a concentration-dependent manner along with improvements in arterial stiffness. These rapid effects (4 h) are lost at 96 h when a relationship between endothelial response and AR polymorphism is found. Potential cardiologic, post-surgical and sports endocrinology implications remain to be fully elucidated. Our results might lead to further studies aimed at evaluating the effects of acute T administration in patients affected with CAD or in post-surgical recovery and, in particular, in elderly men affected by late-onset hypogonadism.
